# The Anti-Diabetic Drug Metformin Rescues Aberrant Mitochondrial Activity and Restrains Oxidative Stress in a Female Mouse Model of Rett Syndrome

**DOI:** 10.3390/jcm9061669

**Published:** 2020-06-01

**Authors:** Ilaria Zuliani, Chiara Urbinati, Daniela Valenti, Maria Cristina Quattrini, Vanessa Medici, Livia Cosentino, Donatella Pietraforte, Fabio Di Domenico, Marzia Perluigi, Rosa Anna Vacca, Bianca De Filippis

**Affiliations:** 1 Department of Biochemical Sciences, Sapienza University of Rome, 00185 Rome, Italy; ilaria.zuliani@uniroma1.it (I.Z.); fabio.didomenico@uniroma1.it (F.D.D.); marzia.perluigi@uniroma1.it (M.P.); 2 Center for Behavioral Sciences and Mental Health, Istituto Superiore di Sanità, 00161 Rome, Italy; chiara.urbinati@iss.it (C.U.); vanessamedici3@gmail.com (V.M.); livia.cosentino@iss.it (L.C.); 3 Institute of Biomembranes, Bioenergetics and Molecular Biotechnologies, National Council of Research, 70126 Bari, Italy; d.valenti@ibbe.cnr.it (D.V.); r.vacca@ibiom.cnr.it (R.A.V.); 4 Core Facilities, Istituto Superiore di Sanità, 00161 Rome, Italy; mariacristina.quattrini@iss.it (M.C.Q.); donatella.pietraforte@iss.it (D.P.)

**Keywords:** Rett syndrome, metformin, repurposing, PGC-1α, Nrf2

## Abstract

Metformin is the first-line therapy for diabetes, even in children, and a promising attractive candidate for drug repurposing. Mitochondria are emerging as crucial targets of metformin action both in the periphery and in the brain. The present study evaluated whether treatment with metformin may rescue brain mitochondrial alterations and contrast the increased oxidative stress in a validated mouse model of Rett syndrome (RTT), a rare neurologic disorder of monogenic origin characterized by severe behavioral and physiological symptoms. No cure for RTT is available. In fully symptomatic RTT mice (12 months old MeCP2-308 heterozygous female mice), systemic treatment with metformin (100 mg/kg ip for 10 days) normalized the reduced mitochondrial ATP production and ATP levels in the whole-brain, reduced brain oxidative damage, and rescued the increased production of reactive oxidizing species in blood. A 10-day long treatment with metformin also boosted pathways related to mitochondrial biogenesis and antioxidant defense in the brain of metformin-treated RTT mice. This treatment regimen did not improve general health status and motor dysfunction in RTT mice at an advanced stage of the disease. Present results provide evidence that systemic treatment with metformin may represent a novel, repurposable therapeutic strategy for RTT.

## 1. Introduction

Metformin is the most widely prescribed treatment for hyperglycemia and type 2 diabetes, even in pediatric patients [1,2]. It belongs to the class of biguanides and acts as a blood glucose-lowering agent by decreasing hepatic gluconeogenesis and improving insulin sensitivity. While metformin has been used to treat diabetes for over 60 years, recent evidences open up the possibility that this clinically approved drug may be repurposed for several other conditions, such as cancer, cardiovascular disorders, neurodegenerative diseases [3], and more recently, neurodevelopmental disorders characterized by intellectual disability [4,5]. Moreover, treatment with metformin was demonstrated to counteract the deleterious effects of aging and provide beneficial effects on mouse healthspan and lifespan [6].

The mechanisms of action of metformin have not been completely clarified. Energy metabolism is a clear focal point of metformin action and emerging evidence suggests that its therapeutic potential may reside in the capacity to target mitochondrial dysfunction [1]. Chronic treatment with metformin in fact improves mitochondrial function and increases antioxidant protection. Consistent with the capability of metformin to pass the blood-brain barrier, these treatment effects have been evidenced both in the periphery and in the brain [7,8,9,10]. The exact nature of the interaction between metformin and mitochondria is still poorly characterized [11,12]. Metformin induces a weak but specific inhibition of the mitochondrial respiratory-chain (MRC) complex I in tissues such as skeletal muscle and liver [13]. Even though complex I is the most studied mitochondrial target of metformin, several signaling pathways relevant for mitochondrial functionality have been found to be impacted by the drug. An emerging target is the peroxisome proliferator-activated receptor gamma coactivator 1-alpha (PGC-1α), a transcriptional coactivator that plays key roles in mitochondrial biogenesis [14]. The beneficial effects of metformin on mitochondrial dysfunction in fibroblasts from Down syndrome (DS) patients were in fact found to be due to the activation of the PGC-1α downstream targets that regulate the transcription of mitochondria-related genes and the activation of the core machinery that governs the dynamic mitochondrial remodeling [5]. Furthermore, metformin increases antioxidant protection through the activation of PGC-1α downstream targets [15,16]. Taken together, these results suggest that treatment with metformin may represent a promising therapeutic strategy to correct mitochondrial dysfunction, even in the brain.

Defective mitochondrial energy production machinery and the resulting increased levels of free radicals are emerging as relevant factors in the pathogenesis of Rett syndrome (RTT) [17], a rare neurological disorder which affects mainly females with an incidence of 1:10,000 live births. After a normal development until 6–18 months of age, patients start showing a progressive loss of previously acquired cognitive, social, and motor skills as a consequence of an early neurological regression. RTT patients may live beyond 50–60 years of age. In the majority of cases (95%), RTT arises from de novo mutations in the X-linked methyl CpG-binding protein 2 (*MECP2*) gene. The encoded MeCP2 protein is expressed ubiquitously but mostly in the brain and it is involved in several processes such as transcriptional regulation, modulation of chromatin structure and RNA splicing [18].

Mitochondrial alterations so far identified in the brain and in hind limb muscle in RTT mouse models include ultrastructural changes, MRC impairment, oxidative phosphorylation (OXPHOS) deficiency and increased reactive oxidizing species (ROS) production [19,20,21,22]. Consistent with data on lymphomonocytes of RTT patients [23,24], mitochondrial dysfunction in RTT mouse brain is accompanied by the downregulation of the protein content of MRC complexes subunits [20] and by reduced energy status in two highly validated RTT mouse models [22]. An impaired antioxidant defense system with high levels of lipid peroxidation and markers of oxidative stress has been also detected both in the blood of RTT patients [25] and in the brain of MeCP2-null mice [26,27]. Furthermore, dyslipidaemia, fatty liver disease, insulin resistance and metabolic syndrome were also uncovered [27,28], confirming that metabolic alterations in RTT patients and mouse models extend beyond mitochondria. 

All these evidences suggest that MeCP2 plays an important role in the regulation of systemic metabolism and *MECP2* mutations provide profound metabolic dysfunctions both at the peripheral and central levels [29]. However, the underlying mechanisms have not been completely clarified and it is currently unclear whether metabolic alterations play a key role in the pathogenesis of RTT. Based on these evidences, the present study addressed whether metformin may rescue brain mitochondrial alterations and contrast the increased oxidative stress in a validated RTT mouse model bearing a MeCP2 truncating mutation (MeCP2-308 mice) [30,31]. We reasoned that, by improving brain mitochondrial dysfunction, metformin may rescue the neurological phenotype, thus representing an innovative and repurposable therapeutic strategy for RTT.

## 2. Experimental Section

### 2.1. Subjects

The experimental subjects were MeCP2-308 heterozygous female (RTT) mice (B6.129S-MeCP2tm1Hzo/J, stock number: 005439; backcrossed to C57BL/6J mice for at least 12 generations from the Jackson Laboratories (USA) and wild-type (WT) littermates. MeCP2-mutated heterozygous females were used as a disease-related model, as they recapitulate the genetic and hormonal milieu of RTT patients [32,33]. Mice were housed in groups of 2-3 in polycarbonate transparent cages (33 × 13 × 14 cm) with sawdust bedding and kept on a 12h light-dark schedule (lights off at 8:00 am). Temperature was maintained at 21 ± 1°C and relative humidity at 60 ± 10%. Animals were provided with tap water ad libitum and a complete pellet diet (Altromin, Germany). Mice were tested at 12 months of age, an age at which they are fully symptomatic [19,34]. All procedures were carried out in accordance with the European Communities Council Directive (10/63/EU) as well as the Italian law (26/2014; approval number by the Italian Ministry for Health: DGSAF 763/2019-PR).

### 2.2. Genotyping

DNA was prepared from a small tail-tip biopsy taken at 21–25 days of age, as previously described [35]. The *MeCP2* alleles were identified by PCR using two sets of primers. Primer set 1 (5′ primer: 5′-AAC GGG GTA GAA AGC CTG-3′ and 3′ primer: 5′- ATG CTC CAG ACT GCC TTG -3′) yields a product of 396 bp identifying the wild-type allele. Primer set 2 (5′ primer: same as for primer set 1 and 3′ primer: 5′- TGA TGG GGT CCT CAG AGC -3′) yields a product of 318 bp identifying the null allele. PCR products were electrophoresed through a 2% NuSieve 3:1 agarose gel (Cambrex Bio Science, Rockland, ME, USA) containing 0.5 μg/mL ethidium bromide and examined under UV light.

### 2.3. Drug Treatment and Experimental Design

Metformin (met-1,1-Dimethylbiguanide) was supplied by Sigma-Aldrich (St Louis, MO, USA) and stored at +4 °C. Metformin was dissolved in saline (sal-0.9% NaCl) and the quantity to be injected daily was calculated according to mouse weight (volume of intraperitoneal injection (ip): 10 mL/kg). RTT mice and WT littermate controls were randomly assigned to receive metformin or sal in a balanced way, according to weight and general health status.

In order to evaluate whether systemic treatment with metformin could rescue brain metabolic alterations and the defective general health status of RTT mice when they present the full symptomatology, on 1-year-old WT and RTT mice, we applied the 10-day long treatment regimen that ameliorates core symptoms in a mouse model of Fragile X [4], a disorder of genetic origin with several symptoms in common with RTT. Metformin was first administered at the dose of 200 mg/kg, according to the protocol described in [4]. However, on the first day of the treatment, 5 out of the 8 mice that received metformin (3 WT and 2 RTT) showed convulsions about an hour after the injection, while one of the experimental subjects did not survive. This prompted us to halve the dose and treat an additional cohort of mice with 100 mg/kg of metformin or saline for 10 days. This new dose was chosen as many studies have already addressed its efficacy [36,37]. Furthermore, a dose response curve has been previously performed [4] that confirms that the concentration levels of metformin that can be achieved with the 100 mg/kg dose are comparable to those obtained with the standard dose used in humans for the treatment of type-2 diabetes (~20 mg/kg), with both giving plasma concentrations in the 10–20 µM range [38].

To assess metformin effects on RTT-related behavioral alterations, experimental mice underwent a test battery 24 hours after the last ip (11^th^ day of the schedule), as previously reported [4]. In particular, we assessed treatment effects on general health status and motor function (open field task and dowel test). At the end of behavioral testing, the experimental mice were sacrificed, and blood and brains were collected for the subsequent mitochondrial and molecular analysis.

### 2.4. Behavioral Assessments

Behavioral testing took place during the dark phase of the light/dark cycle, between 9:00 and 12:00 am, and was carried out by experimenters blind to the mouse genotype and treatment. Mice were experimentally naïve. Mice for each condition were as follows: WT, sal = 8; WT, met = 7; RTT, sal = 8; RTT, met = 8.

#### 2.4.1. Open Field Test

The open field test was performed 24 hours after the last injection to assess locomotor activity, exploratory habits and response to novelty. The apparatus consisted of a black plastic box (40 × 40 cm) enclosed by high walls (35 cm), with brightly illuminated floor (about 13 lux). Each mouse was individually placed inside the arena with its head facing one of the walls and allowed to freely explore the environment during a 10-min session as previously described [34]. The floor of the apparatus was cleaned with 70% ethanol before each testing session. The total distance moved in the arena was automatically detected with specific behavioral tracking software (ANY-maze software Version 4.82, Ugo Basile SRL Gemonio, Italy).

#### 2.4.2. Dowel Test

To evaluate the effects of metformin on motor coordination and balance, the dowel test was performed 5 minutes after the open field test as in [39]. The hardwood round dowel used was 9 mm in diameter and 35 cm long. The dowel was mounted horizontally 50 cm above a 5 cm deep bedding of sawdust. At the beginning of the test, each mouse was placed in the middle of the dowel so that the length of its body was parallel to it. Latency to fall from the dowel was recorded (30 s cut-off). Each mouse repeated the test twice, with an intertrial interval of at least 15 min. If mice were able to walk across and off the dowel, they received the maximum score of 30 s.

#### 2.4.3. General Health Evaluation

The general health of the experimental subjects was qualitatively evaluated by a trained observer, blind to the genotype and treatment of mice as previously described [40], with little modification. The observation of mice was performed on a laboratory bench and the experimental subjects received a score (ranging from 0-normal appearance to 4-highly compromised) for each of these parameters: gait, mobility, breathing, kyphosis, fur, hind limb clasping, tremors, and presence of seizures. The individual scores for each category were subsequently averaged to obtain a semi-quantitative measure of symptom status, called throughout the text “the general health score”. The qualitative evaluation was carried out on the last day of treatment soon after behavioral testing.

### 2.5. Mitochondrial Analyses

At the end of behavioral testing, brains were collected to evaluate whether metformin treatment was able to rescue the reduced mitochondrial ATP production via OXPHOS and restore the alterations in the activity of the MRC complexes and of ATP synthase, the molecular machinery responsible for the majority of cell energy production.

#### 2.5.1. Brain Tissue Dissection and Mitochondria Isolation

The brains of experimental mice (WT, sal; WT, met; RTT, sal; RTT, met; N = 3 per group) were dissected into two hemispheres and cryopreserved in an ice-cold solution consisting of 50 mM K-Mes (pH = 7.1), 3 mM K_2_HPO_4_, 9.5 mM MgCl_2_, 3 mM ATP plus 20% glycerol and 10 mg/mL bovine serum albumin (BSA) and stored at −80 °C until assay. Mitochondria were isolated from cryopreserved tissues by differential centrifugation of brain homogenate as previously described [41] and controls were made for checking mitochondrial integrity and function, as reported in [41].

#### 2.5.2. Mitochondrial ATP Production via OXPHOS

The rate of ATP production by OXPHOS was determined in mitochondria isolated from cryopreserved brain tissues essentially as previously described [42]. Briefly, mitochondria isolated from total brain (0.5 mg protein) were incubated at 37 °C in 2 mL of respiratory medium consisting of 210 mM mannitol, 70 mM sucrose, 20 mM Tris-HCl, 5 mM KH_2_PO_4_/K_2_HPO_4_, (pH = 7.4) plus 5 mg/mL BSA, 3 mM MgCl_2_, in the presence of the ATP detecting system consisting of glucose (2.5 mM), hexokinase (HK, 2 e.u.), glucose 6-phosphate dehydrogenase (G6P-DH, 1 e.u.) and NADP^+^ (0.25 mM) by adding glutamate plus malate (GLU/MAL, 5 mM each) or succinate (SUCC, 5 mM) plus rotenone (ROT, 3 mM), or ascorbate (ASC, 0.5 mM) plus N,N,N′,N′-tetramethyl-p-phenylenediamine (TMPD, 0.25 mM), as energy sources. The reduction of NADP^+^ in the extramitochondrial phase, which reveals ATP formation from externally added ADP (0.5 mM), was monitored as an increase in absorbance at 340 nm. Care was taken to use enough HK/G6P-DH coupled enzymes to ensure a non-limiting ADP-regenerating system for the measurement of ATP production.

#### 2.5.3. Mouse Brain ATP Levels

The brains of experimental subjects (WT, sal; WT, met; RTT, sal; RTT, met; N = 3 per group) were subjected to perchloric acid extraction as described in [43]. In brief, tissues were homogenized in 600 mL of pre-cooled 10% perchloric acid and then centrifuged at 14,000× *g* for 10 min, 4 °C. The amount of tissue ATP was determined enzymatically in KOH-neutralized extracts, as described in [42].

#### 2.5.4. MRC Complex Activities

The MRC complex activities were measured spectrophotometrically in mitochondrial membrane-enriched fractions obtained from mitochondria isolated from cryopreserved brain tissues. For isolation of mitochondrial membrane-enriched fractions, mitochondrial pellets were first frozen at −80 °C, then thawed at 2–4 °C, suspended in 1 mL of 10 mM Tris-HCl (pH 7.5) plus 1 mg/mL BSA and exposed to ultrasound energy for 8 s at 0 °C (11 pulses 0.7 s on, 0.7 s off) at 20 kHz, intensity 2. The ultrasound-treated mitochondria were centrifuged at 600× *g* for 10 min, 4 °C. The supernatant was centrifuged again at 14,000× *g* for 10 min at 4 °C and the resulting pellet was kept at −80 °C until use. Measurement of MRC complex activities was performed essentially as in [44], by two assays which rely on the sequential addition of reagents to measure the activities of complexes I, II, and V (ATPase).

### 2.6. Oxidative Stress Status Analyses

On the blood and the brain of the experimental mice collected 24 hours after the last injection, we also explored metformin treatment effects on the circulating levels of ROS and the oxidative brain damage that occurs in the RTT mouse model.

#### 2.6.1. ROS Levels in Whole Blood by Electron Paramagnetic Resonance (EPR)

Whole blood of the experimental subjects was collected into heparinized tubes at sacrifice to evaluate ROS levels (WT, sal; WT, met; RTT, sal; RTT, met; N = 3–4 per group). The oxidation of the spin probe 1-hydroxy-3-carboxypyrrolidine (CPH, dissolved in degassed phosphate buffer, pH 7.4, and extensively treated with Chelex-100 to avoid metal contamination) to the correspondent 3-carboxy-proxyl radical (CP^•^) [45] was monitored by EPR. The formation of CP^•^ is not specific to a singular oxidant, but it is suitable to screen the totality of ROS (among which O2^•, •^OH, peroxynitrite, transition metal-catalyzed reactions) produced in biological samples. If the intensity of CP^•^ is significantly increased, the presence of a pro-oxidant status is suggested.

Briefly, CPH (0.5 mM) was added to 100 μL whole blood of WT or RTT mice and the intensity of CP^•^ was measured after 20 min at 37 °C. Samples were drawn up into a gas-permeable Teflon tube with 0.81-mm internal diameter and 0.05-mm wall thickness (Zeuss Industrial Products). The Teflon tube was folded four times, inserted into a quartz tube, and fixed to the cavity (4108 TMH) of a Bruker ECS 106 EPR spectrometer equipped with a variable temperature unit (ER4111VT). Spectrometer conditions were: modulation frequency, 100 kHz; microwave frequency, 9.4 GHz; microwave power, 20 mW; gain 1 × 104; modulation amplitude, 1G; conversion time, 20.5 ms; time constant, 82 ms; sweep time, 21 s; and number of scans, 1.

#### 2.6.2. Total 4-hydroxy-2-trans-nonenal Protein Bound (HNE-Adducts) in RTT Mouse Brain

To evaluate protein oxidative damage in RTT mouse hippocampus and metformin treatment effects thereon, we investigated hippocampal levels of HNE-adducts, one of the most abundant and toxic aldehydes generated through ROS-mediated peroxidation of lipids [46]. Hippocampi (WT, sal; WT, met; RTT, sal; RTT, met; N = 9–11 per group) were homogenized and Western blot analyses were performed as described below. The resulting blot was incubated overnight at 4°C with HNE polyclonal antibody (1:2000, Novus Biologicals, Abingdon, UK, #NB100-63093). Next day, the membrane was incubated for 1 h at room temperature with anti-goat horseradish peroxidase-conjugated secondary antibody (A4187, 1:3000) furnished by Sigma-Aldrich (St Louis, MO, USA). The blot was then imaged via the ChemiDoc MP imaging system using Chemiluminescence settings. Subsequent determination of relative abundance via total protein normalization was calculated using Image Lab 6.0 software (Bio-Rad Laboratories Hercules, CA, USA).

### 2.7. Western Blot Analyses

To uncover the molecular mechanisms leading to the improved mitochondrial bioenergetics in metformin treated RTT mouse brain, we next analyzed MRC protein content and the levels of specific targets of metformin by western blotting analysis. To this aim, hippocampi (WT, sal; WT, met; RTT, sal; RTT, met; N = 9–11 per group) were homogenized in RIPA buffer (pH = 7.4) containing 50 mM Tris-HCl (pH = 7.4), 150 mM NaCl, 1% NP-40, 0.25% sodium deoxycholate,1mM EDTA, 0,1% SDS, 1mM PMSF, 1 mM NaF and 1 mM Na3VO4. Brains were homogenized by 20 strokes of a Wheaton tissue homogenizer and centrifuged at 14,000× *g* 4 °C for 10 min to remove cellular debris. Supernatants were collected to determine total protein concentrations by the BCA method (Pierce, Rockford, IL, USA). Western blots were performed as previously described in [47]. Briefly, 15 μg of proteins were separated via SDS-PAGE and transferred to a nitrocellulose membrane by Trans-Blot Turbo Transfer System (Bio-Rad Laboratories Hercules, CA, USA). The blot was imaged by ChemiDoc MP imaging system (Bio-Rad Laboratories Hercules, CA, USA) using the Stain-Free Blot settings. Protein total load captured by Stain-Free Blot technology was later used for total protein normalization. Following, the membrane was blocked with 3% of bovine serum albumin (SERVA Electrophoresis GmbH, Heidelberg, Germany) in TBS solution containing 0.01% Tween 20 and incubated overnight at 4 °C with the following primary antibodies: p^Ser40^Nrf2 (Ab76026, 1:3000), Total OXPHOS (Ab110411, 1:3000) from Abcam (Cambridge, UK), MFN2 (#9482, 1:1000) from Cell Signaling Technology (Danvers, MA, USA), HO-1 (ADI-SPA-895, 1:1000), Nrf2 (ADI-KAP-TF125, 1:1000) from Enzo Life Sciences (Farmingdale, NY, USA), NDUFB8 (NBP2-75586, 1:5000) from Novus Biological, PGC-1α (SC-13067, 1:1000), mtTFA (SC-166965, 1:1000) from Santa Cruz Biotechnology (Palo Alto, CA, USA), OPA1 (612607, 1:1000) from BD Transduction Laboratories (San Jose, CA, USA). Next day, all membranes were incubated for 1 h at room temperature with respective horseradish peroxidase-conjugated secondary antibodies: anti-rabbit (L005661; 1:10000), anti-mouse (L005662; 1:10000) from Bio-Rad Laboratories (Hercules, CA, USA). The blot was then imaged via the ChemiDoc MP imaging system using Chemiluminescence settings. Subsequent determination of relative abundance via total protein normalization was calculated using Image Lab 6.0 software (Bio-Rad Laboratories, Hercules, CA, USA).

### 2.8. Statistical Analyses

Data were analyzed with two-way Analysis of Variance (ANOVA) including genotype (WT vs RTT) and treatment (sal vs met) as independent variables (between-subject factors). Experimental subjects identified as outliers by the use of the Grubbs’ test were excluded from the analyses. *Post hoc* comparisons were performed by Tukey’s test. Results of two-way ANOVA analyses and *post hoc* tests are reported in Supplementary Table S1.

## 3. Results

### 3.1. A 10-Day Long Metformin Treatment at 100 mg/kg Dose Rescues the Aberrant Mitochondrial Bioenergetics in the Brain of RTT Mice

**Figure 1:**
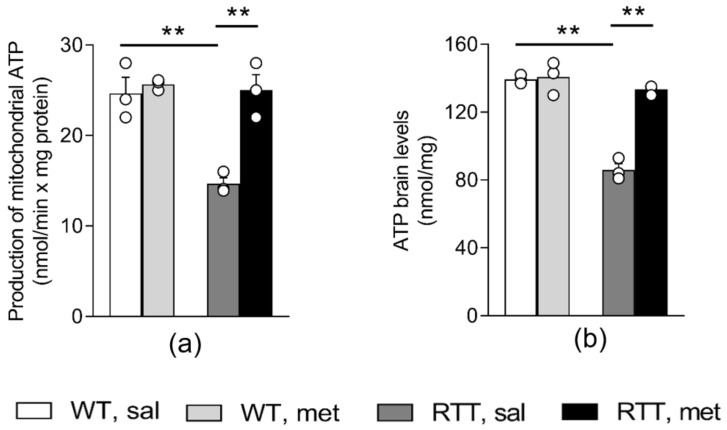
Metformin systemic treatment rescues defective energy status in RTT mouse brain. A 10-day long treatment with metformin (100 mg/kg) completely normalizes the reduced mitochondrial ATP production via oxidative phosphorylation (**a**) and ATP levels (**b**) in RTT mouse brain. N = 3. Data are mean ± SEM. Statistical significance was calculated by two-way ANOVA, with Tukey’s *post hoc* test. ** *p* < 0.01. WT: wild-type mice; RTT: MeCP2-308 heterozygous female mice; sal: saline; met: metformin.

To evaluate if a 10-day long metformin treatment can ameliorate the defective bioenergetic efficiency in RTT mouse brain, mitochondrial ATP synthesis and whole-brain ATP levels were measured. As expected, based on previous reports [19,20], RTT mice showed a significant reduction in mitochondrial ATP synthesis when succinate, the respiratory substrate of complex II, was provided as energy source (p < 0.01; Figure 1a). ATP levels in the whole brain of RTT mice were also decreased in comparison to WT controls ( p < 0.01; Figure 1b). A complete normalization to WT values of both parameters was found in the brain of metformin-injected RTT mice (p < 0.01; Figure 1a,b).

**Figure 2:**
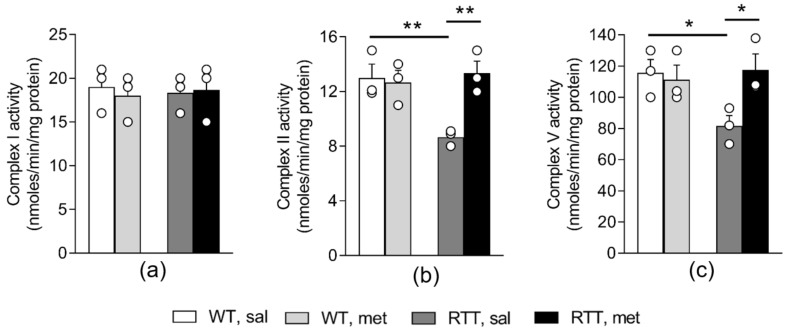
Metformin treatment restores mitochondrial complex II and V activity in the brain of RTT mice. The activity of complex I (**a**) did not differ between the two genotypes and was not affected by the metformin treatment. The activity of mitochondrial respiratory chain complex II (**b**) and complex V (**c**) was reduced in RTT mouse brain compared to WT controls and metformin treatment restored WT-like levels. N = 3. Data are mean ± SEM. Statistical significance was calculated by two-way ANOVA, with Tukey’s *post hoc* test. * *p* < 0.05, ** *p* < 0.01. WT: wild-type mice; RTT: MeCP2-308 heterozygous female mice; sal: saline; met: metformin.

We previously demonstrated that aberrant mitochondrial bioenergetics in the whole brain of RTT mice is accompanied by alterations in the activity of MRC II and V, in the absence of changes in the activity of MRC I, III and IV [19,20]. We thus wondered whether metformin has improved these defects, thus normalizing mitochondrial ATP production in RTT mouse brain. We confirmed a significant reduction in the activity of both complexes in RTT mouse brain compared to WT controls (Complex II: *p* < 0.01; Figure 2b; Complex V: *p* < 0.05; Figure 2c). Metformin treatment increased complex II and V activity thus restoring WT-like levels (*p* < 0.01 and *p* < 0.05; Figure 2b,c). To confirm that metformin treatment does not affect complex I activity in mouse brain [10], we extended the analysis to this complex (Figure 2a). In line with previous studies [19], no significant differences in the activity of complex I were found between WT and RTT mice in whole brain and metformin treatment did not significantly affect this parameter. 

**Figure 3:**
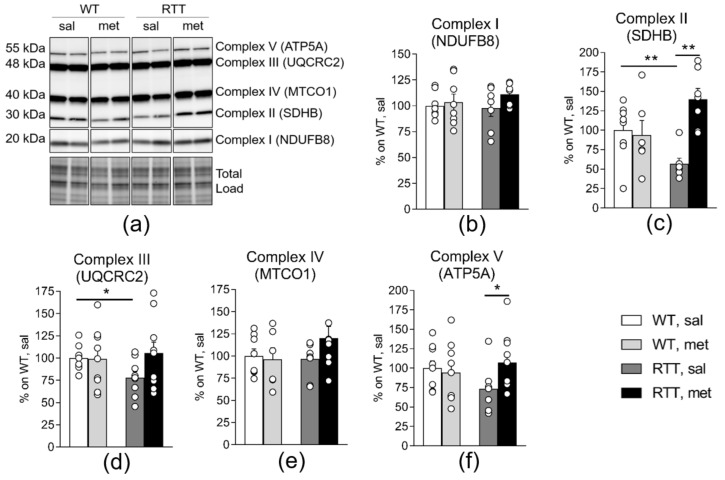
Metformin treatment normalizes decreased oxidative phosphorylation protein content in RTT mouse hippocampus. Protein expression levels of SDHB (subunit of Complex II) (**c**) and UQCRC2 (subunit of Complex III) (**d**) is decreased in the hippocampus of RTT mice. A trend towards a decrease was also observed for ATP5A (subunit of Complex V) (**f**) in RTT compared to WT controls. No genotype differences were found for NDUFB8 (subunit of Complex I) (**b**) and MTCO1 (subunit of Complex IV) (**e**). Metformin treatment normalized protein expression levels of SDHB (subunit of Complex II) (**c**) and ATP5A (subunit of Complex V) (**f**) in RTT mice. Representative blot is shown in (**a**). N = 9–11. Data are mean ± SEM normalized for WT, sal. Statistical significance was calculated by two-way ANOVA, with Tukey’s *post hoc* test. * *p* < 0.05, ** *p* < 0.01. WT: wild-type mice; RTT: MeCP2-308 heterozygous female mice; sal: saline; met: metformin; NDUFB8: NADH:Ubiquinone Oxidoreductase Subunit B8; SDHB: Succinate Dehydrogenase Complex Iron Sulfur Subunit B; UQCRC2: Ubiquinol-Cytochrome C Reductase Core Protein II; MTCO1: mitochondrially encoded cytochrome c oxidase I; ATP5A: ATP Synthase, H^+^ Transporting, Mitochondrial F1 Complex.

Previous data demonstrated that MRC dysfunctions are accompanied by a reduction of specific complex subunits protein levels in mitochondria isolated from RTT mouse cortex and hippocampus [19]. A similar profile, even though less pronounced, was found in RTT homogenized hippocampi. A significant reduction of SDHB (Complex II: 45% of WT; *p* < 0.01; Figure 3c) and of UQCRC2 (Complex III: 24% of WT; *p* < 0.05; Figure 3d) were in fact evident in RTT compared to WT group. ATP5A levels (Complex V) appeared reduced as well (27% of WT). This genotype difference did however miss statistical significance (*p* = 0.146; Figure 3f). No genotype differences were observed concerning the levels of NDUFB8 (complex I) and MTCO1 (complex IV) (Figure 3b,e) in mouse hippocampus. Importantly, metformin treatment significantly increased the protein content of the MRC complexes whose activity is defective in RTT mouse brain (Figure 3c,f, SDHB (complex II) and ATP5A (complex V): *p* < 0.01 and *p* < 0.05), thus restoring WT-like levels. No significant treatment effects were found for the other MRC complexes. Metformin treatment thus normalized both the activity and the complex subunits protein levels of complex II and V in RTT mouse brain.

### 3.2. RTT Female Mice Show an Increased Oxidative Stress Status that Is Restored by Metformin both in the Brain and in the Blood

We next considered whether the treatment with metformin can rescue the aberrant oxidative stress status that occurs in RTT [25,48,49].

**Figure 4:**
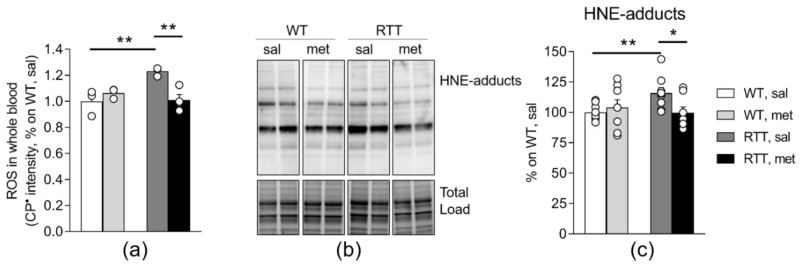
Metformin treatment rescues increased oxidative stress status in the brain and in the blood of RTT mice. (**a**) Blood ROS levels, measured as the intensity of formation of CP^•^ by EPR, were significantly higher in RTT, sal mice compared to WT controls, confirming the occurrence of a pro-oxidant status in RTT mice. Metformin normalized this value in whole blood of RTT mice to the same level of WT controls. N = 3–4. One subject of the experimental group RTT, sal was identified as outlier and thus, excluded from the analysis. (**b–c**) Metformin treatment reduces the abnormal accumulation of protein 4-hydroxynonenal (HNE) adducts in RTT mouse hippocampus (**c**). Representative blot is shown in panel (**b**). N = 9–11. Data are mean ± SEM normalized for WT, sal. Statistical significance was calculated by two-way ANOVA, with Tukey’s *post hoc* test. * *p* < 0.05, ** *p* < 0.01. WT: wild-type mice; RTT: MeCP2-308 heterozygous female mice; sal: saline; met: metformin; HNE-adducts: 4-hydroxy-2-trans-nonenal protein bound.

Blood ROS levels, measured as the intensity of formation of CP^•^ by EPR, were significantly higher in RTT mice compared to WT controls (*p* < 0.01; Figure 4a), confirming the occurrence of a pro-oxidant status in RTT mice [25,48,49]. Interestingly, metformin treatment decreased the intensity of CP^•^ selectively in the whole blood of RTT mice and normalized this value to the same level of WT controls (*p* < 0.01; Figure 4a).

In conditions of oxidative stress, proteins are highly vulnerable, and may be the target of a number of modifications that affects their functions [50]. Formation of adducts with lipid peroxidation can be detected as index of tissue-specific damage. Consistently, we found an increase of protein-HNE-adducts in RTT mouse hippocampus compared to WT controls (*p* < 0.01; Figure 4c), confirming the occurrence of brain oxidative damage in RTT [26]. Such an increase of protein oxidation was rescued by metformin treatment (*p* < 0.05; Figure 4c).

### 3.3. Metformin Systemic Treatment Boosts Pathways Related to Mitochondrial Biogenesis and Remodelling in the Brain of RTT Mice

**Figure 5:**
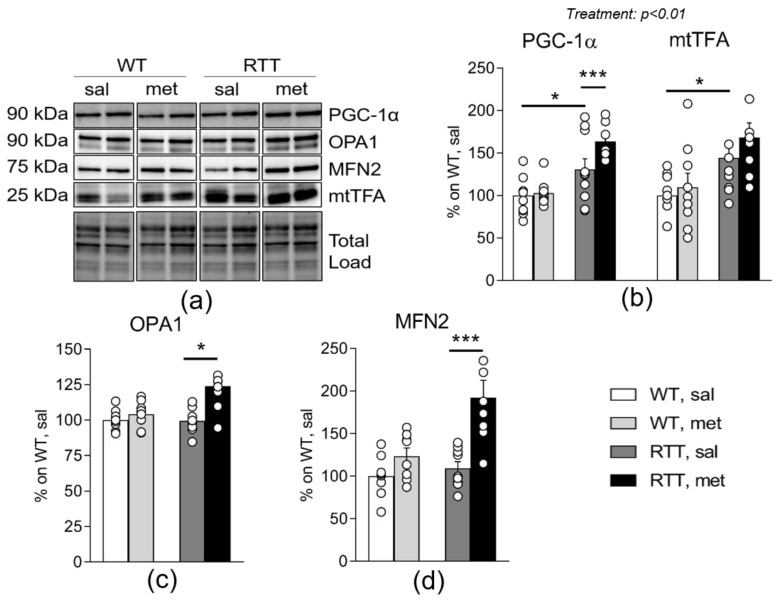
Metformin systemic treatment boosts pathways related to mitochondrial biogenesis and remodeling in RTT mouse brain. PGC-1α protein expression and the levels of its downstream target mtTFA are increased in RTT mouse hippocampus compared to WT controls (**b**). Metformin treatment exacerbates this genotype difference. Following metformin treatment, RTT mouse hippocampus shows an increase in mitochondrial biogenesis related protein OPA1 (**c**) and MFN2 (**d**). Representative blots are reported in (**a**). N = 9–11. Data are mean ± SEM normalized for WT, sal. Statistical significance was calculated by two-way ANOVA, with Tukey’s *post hoc* test. * *p* < 0.05; *** *p* < 0.001. WT: wild-type mice; RTT: MeCP2-308 heterozygous female mice; sal: saline; met: metformin; PGC-1α: peroxisome proliferator-activated receptor gamma coactivator 1-alpha; mtTFA: Mitochondrial transcription factor A; OPA1: GTPase Optic Atrophy 1; MFN2: GTPases Mitofusin 2.

To shed light on the mechanisms leading to the beneficial effects of metformin on brain mitochondrial alterations, we analyzed the expression of PGC-1α and of its downstream target transcription factor A (mtTFA), transcriptional coactivators of mitochondria-related genes [51] that play key roles in mitochondrial biogenesis [14]. We focused on this signaling pathway, based on previous studies demonstrating that metformin promotes its activation [14]. Intriguingly, we found an increase of PGC-1α protein expression levels in the hippocampus of RTT mice compared to WT (*p* < 0.05; Figure 5b) and a corresponding increase of its downstream target mtTFA (*p* < 0.05; Figure 5b). Metformin treatment exacerbated both PGC-1α protein levels (*p* < 0.001; Figure 5b) as well as protein expression of its target mtTFA (treatment: *p* = 0.003). 

Metformin has been also demonstrated to activate the core machinery that governs the dynamic mitochondrial remodeling, through the induction of proteins that regulate fusion, namely the inner mitochondrial membrane GTPase Optic Atrophy 1 (OPA1) and the outer mitochondrial membrane fusion GTPases Mitofusin 2 (MFN2) [5]. We found that both OPA1 and MNFN2 protein levels were increased selectively in the hippocampus of metformin-treated RTT mice compared to saline groups (OPA1: *p* < 0.05; Figure 5c; MFN2: *p* < 0.001; Figure 5d).

### 3.4. Metformin Systemic Treatment Boosts Pathways Related to the Antioxidant Response in the Brain of RTT Mice

**Figure 6:**
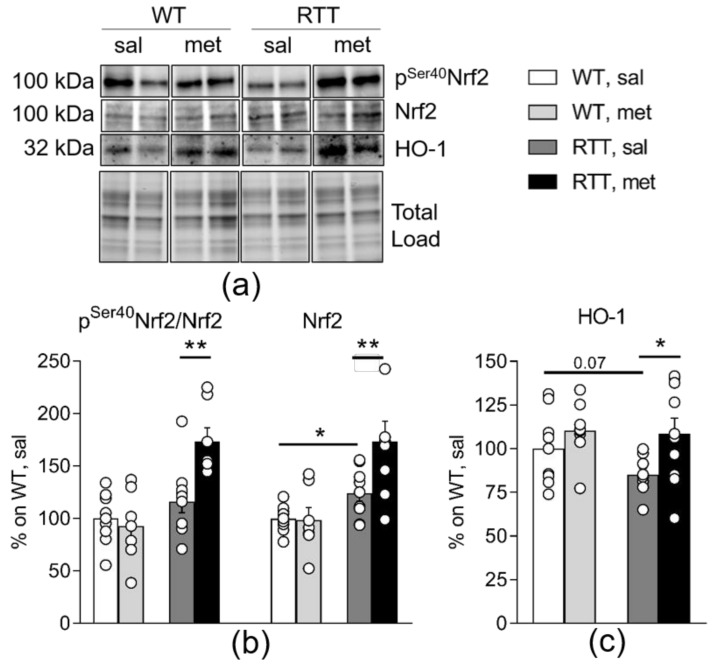
Metformin systemic treatment boosts pathways related to antioxidant response in RTT mouse brain. Metformin treatment induces an antioxidant response selectively in the hippocampus of RTT mice through the increase of Nrf2 protein expression and activation (**b**), and the corresponding increase in Nrf2 downstream target protein HO-1 (**c**). N = 9–11. Representative blot is included in (**a**). Data are mean ± SEM normalized for WT, sal. Statistical significance was calculated by two-way ANOVA, with Tukey’s *post hoc* test. * *p* < 0.05; ** *p* < 0.01. WT: wild-type mice; RTT: MeCP2-308 heterozygous female mice; sal: saline; met: metformin; Nrf2: nuclear respiratory factor 2; HO-1: heme oxygenase-1.

Consistent with its capacity to increase antioxidant protection, metformin also activates the nuclear respiratory factor 2 (Nrf2), a PGC-1α downstream target that increases the expression of the antioxidant protein heme oxygenase-1 (HO-1) and plays a crucial role in defense mechanisms against cellular oxidative stress [15,16,52]. To verify whether activation of this signaling pathway may account for metformin beneficial effects on the increased oxidative stress status in RTT, we analyzed total levels of Nrf2 and of HO-1 in the hippocampus of the experimental mice. Phosphorylation of Nrf2 at Ser40 was also evaluated as an index of its activation status. In fact, under physiological conditions Nrf2 is maintained in an inactive state by Kelch ECH associating protein 1 (Keap1), but when phosphorylated Nrf2 separates from Keap1 and translocates into the nucleus to induce transcription of HO-1 and other antioxidant factors. We found that treatment with metformin induced a consistent increase of Nrf2 expression in RTT mice (*p* < 0.01; Figure 6b) and, more importantly, it also promoted its phosphorylation (*p* < 0.01; Figure 6b). The increased phosphorylation of Nrf2 was accompanied by an increased transcription and subsequent translation of HO-1 in metformin-treated RTT mouse brain (*p* < 0.05; Figure 6c), thus confirming enhanced metformin-induced Nrf2 nuclear import in RTT mouse hippocampus.

### 3.5. A 10-Day Long Treatment with Metformin at 100 mg/kg Dose Does Not Improve Behavioral Alterations in RTT Female Mice at an Advanced Stage of the Disease

To assess whether the metformin treatment could rescue motor dysfunction and the compromised general health status of RTT female mice, a battery of behavioral tests was carried out 24 hours after the last systemic treatment with metformin or saline. 

**Figure 7:**
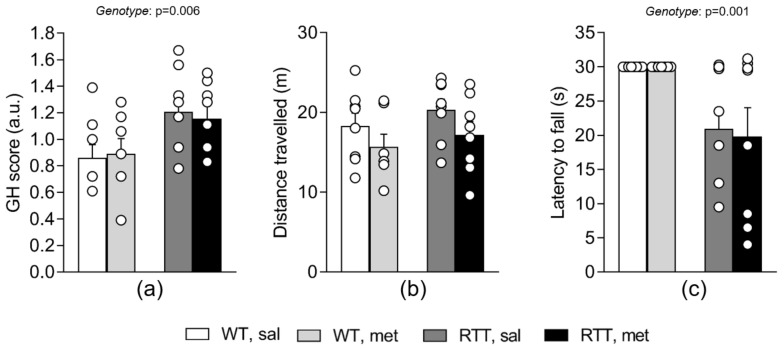
A 10-day long treatment with metformin does not affect the compromised general health (GH) status and motor dysfunction of RTT mice. RTT female mice received higher scores compared to WT mice in the GH evaluation (**a**), confirming the presence of phenotypic alterations. No treatment effects were found on this parameter. Total distance moved in the open field test (**b**) does not significantly differ among groups. RTT mice displayed shorter latencies to fall compared to WT controls in the dowel test (**c**). The metformin treatment did not affect the motor performance of the experimental subjects. N = 7–8. Data are mean ± SEM. Statistical significance was calculated by two-way ANOVA. WT: wild-type mice; RTT: MeCP2-308 heterozygous female mice; sal: saline; met: metformin; GH: general health.

The evaluation of the general health status of the experimental mice confirmed that RTT mice show significant phenotypic alterations compared to WT controls (Figure 7a, genotype: *p* = 0.006). The metformin treatment did not affect these parameters in either WT or RTT mice (Figure 7a). The open field and the dowel tests were applied to assess locomotor activity and coordination and balance, respectively. No significant differences in locomotion were highlighted among groups in the open field test, measured as the total distance travelled (Figure 7b). However, all the experimental mice treated with metformin tended to move less in the arena compared to those treated with saline. This difference just missed the statistical significance (Figure 7b). An impaired motor coordination of RTT female mice was confirmed in the dowel test. Indeed, RTT mice fell off the dowel earlier compared with WT mice (Figure 7c). Metformin treatment did not exert any effect on the motor coordination of the experimental subjects (Figure 7c).

## 4. Discussion

The present study evaluated the possibility to repurpose the anti-diabetic drug metformin for RTT, a severe neurologic disorder characterized by behavioral, physiological as well as metabolic alterations, for which no cure currently exists. Present findings provide innovative evidence that alterations in brain mitochondrial bioenergetics, the oxidative stress status, and the antioxidant defense can be profoundly improved by metformin in a female mouse model of RTT at an advanced stage of the disease.

Increasing evidence demonstrates that besides its well-known antihyperglycemic action, metformin exerts pleiotropic effects, providing a general improvement of cellular energetics, even in the brain [1]. Emerging evidence also points to mitochondria as crucial targets of this clinically approved drug [11,12]. These observations have attracted much attention since they have highlighted the possibility to extend the use of metformin to pathologies other than diabetes, including neurologic disorders [4,53,54,55]. Present findings lie in this direction suggesting that metformin represents an intriguing candidate drug even for RTT. Recent studies in fact support metabolic alterations as key components of RTT pathogenesis, with mouse models and patients showing numerous alterations ranging from lipid metabolism perturbation to mitochondrial abnormalities [27,29]. The therapeutic potential for RTT of drugs targeting metabolic dysfunction is however still unclear [29]. We have previously demonstrated that brain mitochondrial dysfunction in RTT can be rescued by targeting the Rho GTPases family of proteins and this effect is accompanied by a significant improvement of neurobehavioral alterations [19,56], suggesting that mitochondria may represent a promising therapeutic target. The applicability in the clinical setting of these molecules is however still under investigation. In this context, we aimed at identifying clinically approved drugs targeting brain mitochondria dysfunction that could be promptly repurposed, thus increasing the translational value of this pharmacological approach. 

In spite of the promising results suggesting the therapeutic efficacy of metformin for a plethora of disorders, the molecular details of its mechanism of action are not fully elucidated. Given that transcriptional defects of mitochondrial genes have been demonstrated to occur in RTT, that may account for mitochondrial dysfunction [24,49], it is reasonable to hypothesize that the restoration of the reduced protein content and of the activity of MRC complexes by metformin might have rescued mitochondrial bioenergetics alterations in RTT mouse brain. These effects may be achieved through a compensatory boost of the PGC-1α signaling [14,57]. Metformin in fact promoted, selectively in RTT mouse hippocampus, a transcriptional up-regulation of PGC-1α downstream target proteins known to stimulate either mitochondrial biogenesis or the organelle remodeling. Interestingly, a similar effect has been previously reported in fibroblasts from DS patients [5]. Of note, however, a constitutive downregulation of PGC-1α ensues in this cellular model of DS, that has been suggested to contribute to the aberrant mitochondrial functionality. An opposite profile was found in RTT mouse hippocampus, where a reduced protein content of MRC complexes subunits was accompanied by an increase of PGC-1α protein expression levels and a corresponding increase of its downstream target mtTFA, an activator of mitochondrial genes transcription. These results suggest that the induction of mitochondrial biogenesis by PGC-1α may not be sufficient (or sufficiently efficient) to compensate for the bioenergetic alterations in RTT mouse brain. Intriguingly, we found that metformin treatment strongly exacerbated the increase of PGC-1α and mtTFA protein expression levels in RTT mouse brain. Whether these molecular effects may have contributed to the beneficial effects of metformin on RTT-related aberrant mitochondrial functionality certainly needs further investigations. Interestingly, metformin treated RTT mice also showed an induction in the brain expression of OPA1 and MFN2, proteins that regulate dynamic mitochondrial remodeling, suggesting that the simultaneous effect on mitochondrial biogenesis and the organelle remodeling, the two leading mechanisms regulating mitochondrial functionality, may be the key mechanism underlying the beneficial effects of metformin. Further studies addressing treatment effects on the quantity and morphology of mitochondria in RTT mouse brain may help to clarify the actual contribution of the reported molecular effects. 

Besides the effects on mitochondrial functionality, one of the key results of this study is the metformin-induced amelioration of the aberrant prooxidant status and of the brain oxidative damage in RTT. Available data suggest that the main mechanism by which metformin reduces mitochondrial ROS production and improves oxidative stress status is a mild and selective inhibition of the MRC complex I [58]. Nevertheless, the impact that metformin has on cellular energy homeostasis is tissue-specific and the investigation of its action in the brain is far from complete and still a challenge in biguanide research [1]. Herein, we demonstrate that metformin had no effects on complex I activity in mouse brain. We did however find that metformin stimulates the PGC-1α/Nrf2/HO-1 signaling pathway selectively in RTT mouse brain. Indeed, previous works suggested that alterations in the activation of Nrf2 may be responsible for the increased oxidative stress in RTT [59]. In fact, the stimulation of PGC-1α/Nrf2 pathway is known to boost the expression of fundamental mitochondrial antioxidant genes, preventing oxidative injury and mitochondrial dysfunction [60]. Present results provide support to this hypothesis by demonstrating that stimulation of the expression and nuclear import of Nrf2 by metformin and the consequent transcription of genes that regulate the antioxidant defense, such as HO-1, selectively in RTT mouse brain was accompanied by an improvement of the oxidative stress status. 

Interestingly, metformin treatment did not boost pathways related to mitochondrial biogenesis and antioxidant response in WT mice. Indeed, previous studies have demonstrated that metformin’s effects on mitochondrial dynamics are strictly dependent on the initial bioenergetic status [8]. How metformin adapts its effects is however still unclear. Our results lie in the same direction and suggest that PGC-1α dependent pathways may play a crucial role in mediating metformin differential effects on bioenergetic alterations.

Importantly, acute injections of the 200 mg/kg dose of metformin in our study did induce convulsions, thus leading us to test the efficacy of the 100 mg/kg dose. The higher initial dose of metformin was chosen as it provides beneficial effects in a mouse model of Fragile X [4], a disorder with many symptoms in common with RTT. As the main difference from the Fragile X study concerns the age of the animals (two months vs about 1 year in the present study), it is conceivable that the 200 mg/kg may have provided convulsions in both WT and RTT mice due to their advanced age. Consistently, previous studies have demonstrated that metformin exerts different effects depending on the age of the mice [8]. Furthermore, Martin-Montalvo and colleagues [6] similarly observed that chronic high doses of metformin in old mice can be toxic. Present results stress the need to further investigate metformin dose-dependent effects during aging. 

Contrary to our expectations, metformin induced improvements in mitochondrial bioenergetics and oxidative stress status were not accompanied by the rescue of health conditions and motor dysfunction in RTT mice. Present data may challenge the pathogenic role of mitochondrial dysfunction for RTT and suggest that targeting brain mitochondrial dysfunction may not be the correct therapeutic strategy for this disorder. We cannot however exclude that the lack of beneficial effects on the behavioral phenotype of a 10-day long treatment with metformin in RTT mice may be due to the short duration of the treatment or the advanced age of the experimental subjects. Several studies have in fact demonstrated that mitochondrial dysfunction may precede the establishment of behavioral symptoms [50]. Given the essential role of mitochondrial function in mediating neurogenesis and neural circuits rearrangement [61,62], it is conceivable that an early treatment with metformin could rescue the aberrant neurodevelopment in RTT mice and may produce better outcomes on RTT-related behavioral alterations. It is also plausible that the restoration of aberrant brain bioenergetics and oxidative status for more than 10 days may be needed for a neurologic improvement to occur at a fully symptomatic stage of the disease. 

Normalization of impaired performances represents the most attractive outcome in a translational context. Further studies are thus certainly needed aimed at testing whether a pharmacological intervention with metformin during specific time windows of neurodevelopment or a longer administration schedule once symptoms are already established can prevent disease onset or provide beneficial effects on the neurobehavioral alterations in RTT mice. Since accumulated damage to mitochondria acts as a key factor underlying cognitive deficits which are reported as one of the most debilitating symptoms in RTT, a more in-depth examination of metformin effects on learning and memory is also needed to completely verify metformin efficacy.

**Figure 8:**
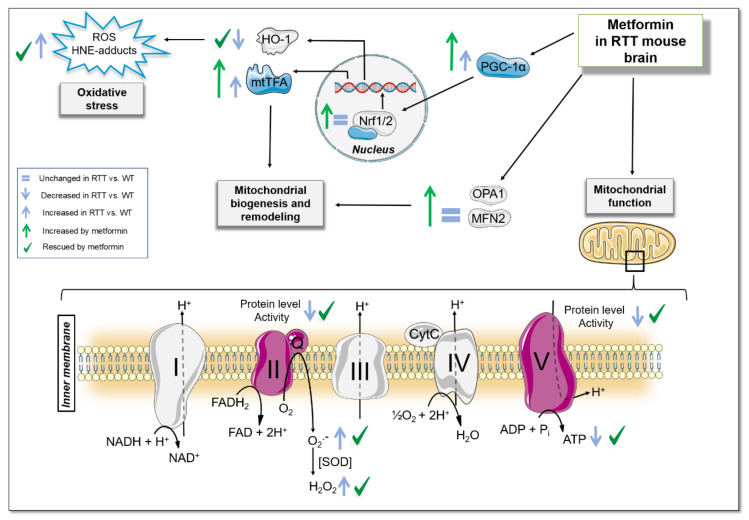
Summary Diagram. A 10-day long treatment with metformin normalizes the reduced mitochondrial ATP production and ATP levels in whole brain of a validated RTT mouse model at an advanced stage of the disease. This is achieved through the restoration of the defective activity of mitochondrial respiratory chain complex II and V and of their reduced protein content. These beneficial effects are accompanied by a strong activation of signaling pathways related to both mitochondrial biogenesis and remodeling (PGC-1α/mtTFA, OPA1 and MFN2) selectively in RTT mouse brain. Metformin also ameliorates prooxidant status and brain oxidative damage in RTT brain, through the stimulation of Nrf2 signaling and the consequent transcription of antioxidant genes such as HO-1. WT: wild-type mice; RTT: MeCP2-308 heterozygous female mice; HNE-adducts: 4-hydroxy-2-trans-nonenal protein bound; PGC-1α: peroxisome proliferator-activated receptor gamma coactivator 1-alpha; mtTFA: Mitochondrial transcription factor A; OPA1: GTPase Optic Atrophy 1; MFN2: GTPases Mitofusin 2; ROS: reactive oxidizing species.

Taken together, present results provide preclinical evidence that significant improvements in mitochondrial dysfunction and oxidative stress status alterations can be achieved in fully symptomatic RTT mice by treatment with metformin (Figure 8), a drug which could be promptly repurposed, even in pediatric patients [63]. Even though further studies are needed to uncover the underlying molecular mechanisms and the most suitable time window for metformin administration, this study suggests that RTT patients may benefit from metformin administration, thus opening a new window of therapeutic opportunities for this severe and untreatable disorder.

## Supplementary Materials

The following are available online at https://www.mdpi.com/2077-0383/9/6/1669/s1, Table S1: Summary of two-way ANOVA analyses and *post hoc* tests of all the results, Figure S1: Full Immunoblot of Western blot results.
