# Supplementary material for: The Anti-Diabetic Drug Metformin Rescues Aberrant Mitochondrial Activity and Restrains Oxidative Stress in a Female Mouse Model of Rett Syndrome
Source: J Clin Med. 2020 Jun 1;9(6):1669. doi: 10.3390/jcm9061669 (PMC7355965; doi:10.3390/jcm9061669)
Supplement: Supplementary file 1 [file jcm-09-01669-s001.pdf]

Supplementary information

Table S1 – Summary of two-way ANOVA analyses and *post hoc* tests of all the results.

Target	Genotype	Treatment	Genotype*Treatment Interaction	*Post hoc* WT,sal VS RTT,sal	*Post hoc* RTT,sal VS RTT,met
Mitochondrial ATP production	**$F_{(1,8)}$=17.067; p=0.003**	**$F_{(1,8)}$=19.267; p=0.003**	**$F_{(1,8)}$=13.067; p=0.007**	**p<0.01	**p<0.01
ATP (brain levels)	**$F_{(1,8)}$=74.604; p<0.001**	**$F_{(1,8)}$=48.009; p=0.001**	**$F_{(1,8)}$=42.892; p=0.002**	**p<0.01	**p<0.01
Complex I (activity)	$F_{(1,8)}$=0.000	$F_{(1,8)}$=0.047; p=0.835	$F_{(1,8)}$=0.186; p=0.677	NS	NS
Complex II (activity)	$F_{(1,8)}$=5.042; p=0.055	**$F_{(1,8)}$=7.042; p=0.029**	**$F_{(1,8)}$=9.375; p=0.016**	**p<0.01	**p<0.01
Complex V (activity)	$F_{(1,8)}$=2.458; p=0.156	$F_{(1,8)}$=3.220; p=0.110	$F_{(1,8)}$=5.223; p=0.052	*p<0.05	*p<0.05
Complex I – NDUFB8 (protein expression levels)	$F_{(1,32)}$=0.210; p=0.650	$F_{(1,32)}$=1.821; p=0.188	$F_{(1,32)}$=0.661; p=0.423	NS	NS
Complex II – SDHB (protein expression levels)	**$F_{(1,32)}$=8.098; p=0.009**	$F_{(1,32)}$=0.015; p=0.903	**$F_{(1,32)}$=10.99; p=0.003**	**p<0.01	**p<0.01
Complex III – UQCRC2 (protein expression levels)	$F_{(1,32)}$=2.024; p=0.164	$F_{(1,32)}$=0.685; p=0.414	$F_{(1,32)}$=2.247; p=0.143	*p<0.05	NS
Complex IV – MTCO1 (protein expression levels)	$F_{(1,32)}$=0.806; p=0.377	$F_{(1,32)}$=0.889; p=0.354	$F_{(1,32)}$=1.582; p=0.219	NS	NS
Complex V – ATP5A (protein expression levels)	$F_{(1,32)}$=2.892; p=0.099	$F_{(1,32)}$=1.043; p=0.315	**$F_{(1,32)}$=0.496; p=0.033**	NS	*p<0.05
ROS (blood levels)	$F_{(1,9)}$=4.972; p=0.053	$F_{(1,9)}$=3.885; p=0.080	**$F_{(1,9)}$=12.732; p=0.006**	**p<0.01	**p<0.01
HNE-adducts (protein expression levels)	$F_{(1,32)}$=1.844; p=0.185	$F_{(1,32)}$=1.696; p=0.203	**$F_{(1,32)}$=5.153; p=0.031**	**p<0.01	*p<0.05
PGC-1α (protein expression levels)	**$F_{(1,32)}$=5.349; p=0.027**	**$F_{(1,32)}$=29.99; p<0.001**	$F_{(1,32)}$=2.111; p=0.156	*p<0.05	***p<0.001
mtTFA (protein expression levels)	$F_{(1,32)}$=1.175; p=0.287	**$F_{(1,32)}$=11.05; p=0.003**	$F_{(1,32)}$=0.212; p=0.648	*p<0.05	NS
OPA1 (protein expression levels)	**$F_{(1,32)}$=4.225; p=0.047**	$F_{(1,32)}$=3.034; p=0.090	$F_{(1,32)}$=0.162; p=0.213	NS	*p<0.05
MFN2 (protein expression levels)	**$F_{(1,32)}$=18.12; p=0.002**	**$F_{(1,32)}$=11.45; p=0.002**	**$F_{(1,32)}$=5.357; p=0.028**	NS	***p<0.001
p^{Ser40}Nrf2/Nrf2 (protein activation levels)	**$F_{(1,32)}$=5.022; p=0.033**	**$F_{(1,32)}$=18.86; p=0.002**	**$F_{(1,32)}$=8.328; p=0.007**	NS	**p<0.01
Nrf2 (protein expression levels)	$F_{(1,32)}$=3.631; p=0.067	**$F_{(1,32)}$=15.49; p=0.005**	$F_{(1,32)}$=4.050; p=0.054	*p<0.05	**p<0.01
HO-1 (protein expression levels)	**$F_{(1,32)}$=6.617; p=0.015**	$F_{(1,32)}$=1.598; p=0.215	$F_{(1,32)}$=0.976; p=0.330	NS	*p<0.05
GH score (General health evaluation)	**$F_{(1,27)}$=8.716; p=0.006**	$F_{(1,27)}$=0.010; p=0.922	$F_{(1,27)}$=0.133; p=0.718	NS	NS
Distance travelled (Open field test)	$F_{(1,27)}$=1.289; p=0.266	$F_{(1,27)}$=3.453; p=0.074	$F_{(1,27)}$=0.031; p=0.860	NS	NS
Latency to fall (Dowel test)	**$F_{(1,27)}$=13.156; p=0.001**	$F_{(1,27)}$=0.055; p=0.817	$F_{(1,27)}$=0.055; p=0.817	NS	NS

The main effects of genotype and treatment as well as their interactions of all the analyses included in the present article were reported. For each factor there were two levels (Genotype: WT, RTT; Treatment: sal, met). Abbreviations. WT: wild-type mice; RTT: MeCP2-308 heterozygous female mice; sal: saline; met: metformin; NDUFB8: NADH:Ubiquinone Oxidoreductase Subunit B8; SDHB: Succinate Dehydrogenase Complex Iron Sulfur Subunit B; UQCRC2: Ubiquinol-Cytochrome C Reductase Core Protein II; MTCO1: mitochondrially encoded cytochrome c oxidase I; ATP5A: ATP Synthase, H^+ Transporting, Mitochondrial F1 Complex; HNE-adducts: 4-hydroxy-2-trans-nonenal protein bound; PGC-1α: peroxisome proliferator-activated receptor gamma coactivator 1-alpha; mtTFA: Mitochondrial transcription factor A; OPA1: GTPase Optic Atrophy 1; MFN2: GTPases Mitofusin 2; Nrf2: nuclear respiratory factor 2; HO-1: heme oxygenase-1; GH score: general health score; NS: Not Significant.

FigureS1:

(a)

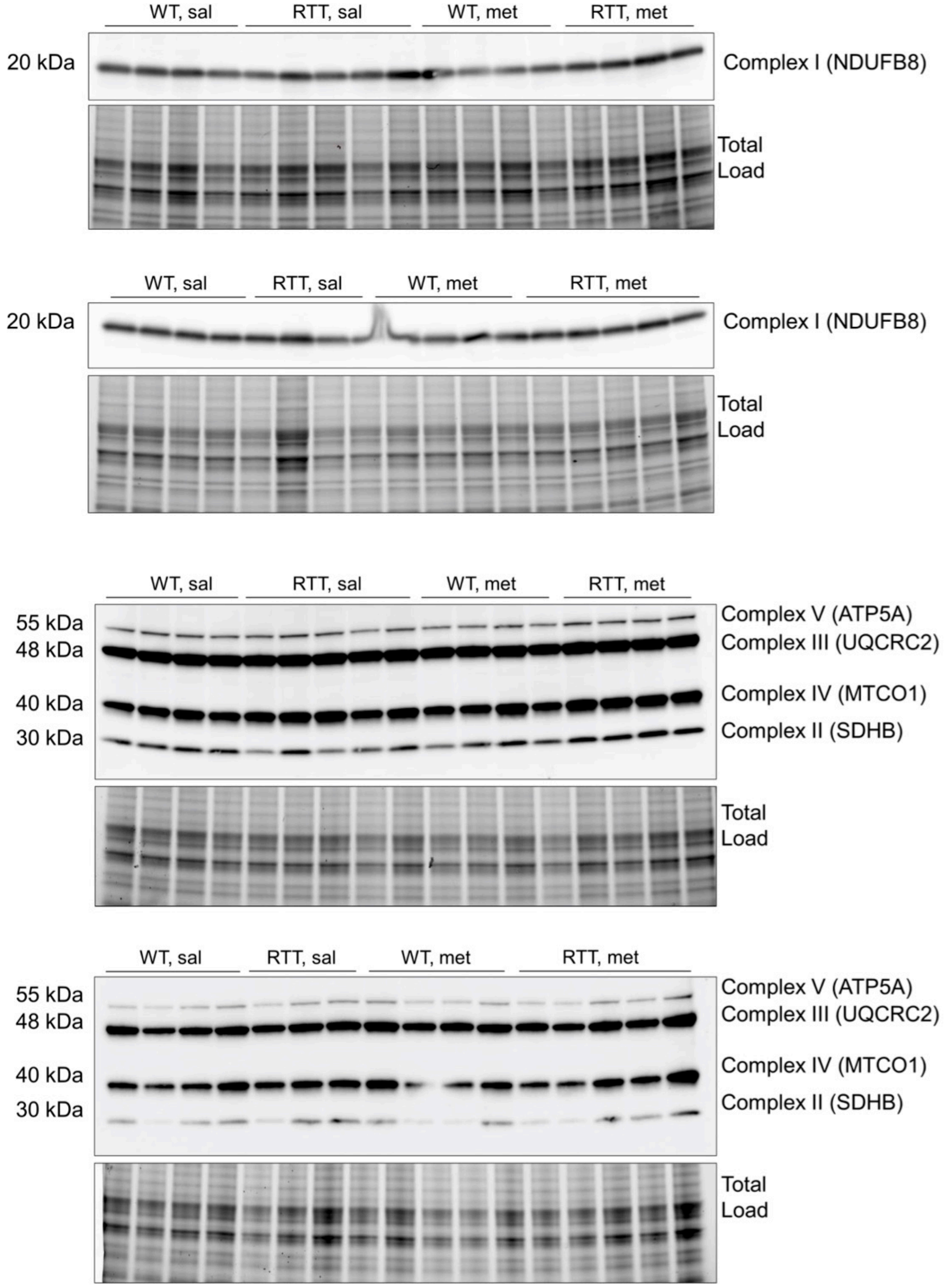
WT, sal
RTT, sal
WT, met
RTT, met
20 kDa
Complex I (NDUFB8)
Total
Load
WT, sal
RTT, sal
WT, met
RTT, met
20 kDa
Complex I (NDUFB8)
Total
Load
WT, sal
RTT, sal
WT, met
RTT, met
55 kDa
48 kDa
40 kDa
30 kDa
Complex V (ATP5A)
Complex III (UQCRC2)
Complex IV (MTCO1)
Complex II (SDHB)
Total
Load
WT, sal
RTT, sal
WT, met
RTT, met
55 kDa
48 kDa
40 kDa
30 kDa
Complex V (ATP5A)
Complex III (UQCRC2)
Complex IV (MTCO1)
Complex II (SDHB)
Total
Load

(b)

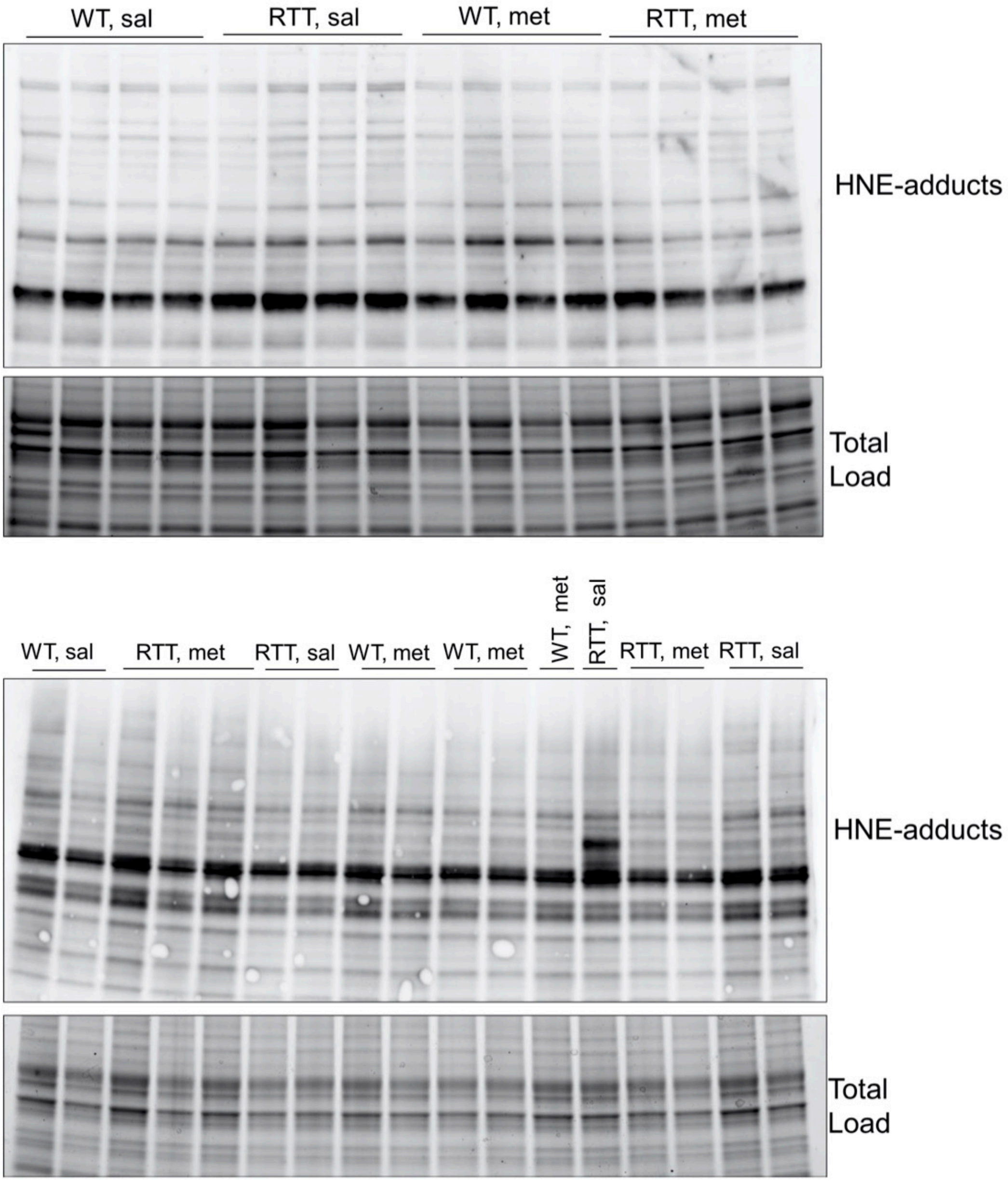
WT, sal
RTT, sal
WT, met
RTT, met
HNE-adducts
Total Load
WT, sal
RTT, met
RTT, sal
WT, met
WT, met
WT, met
RTT, sal
RTT, met
RTT, sal
HNE-adducts
Total Load

(c)

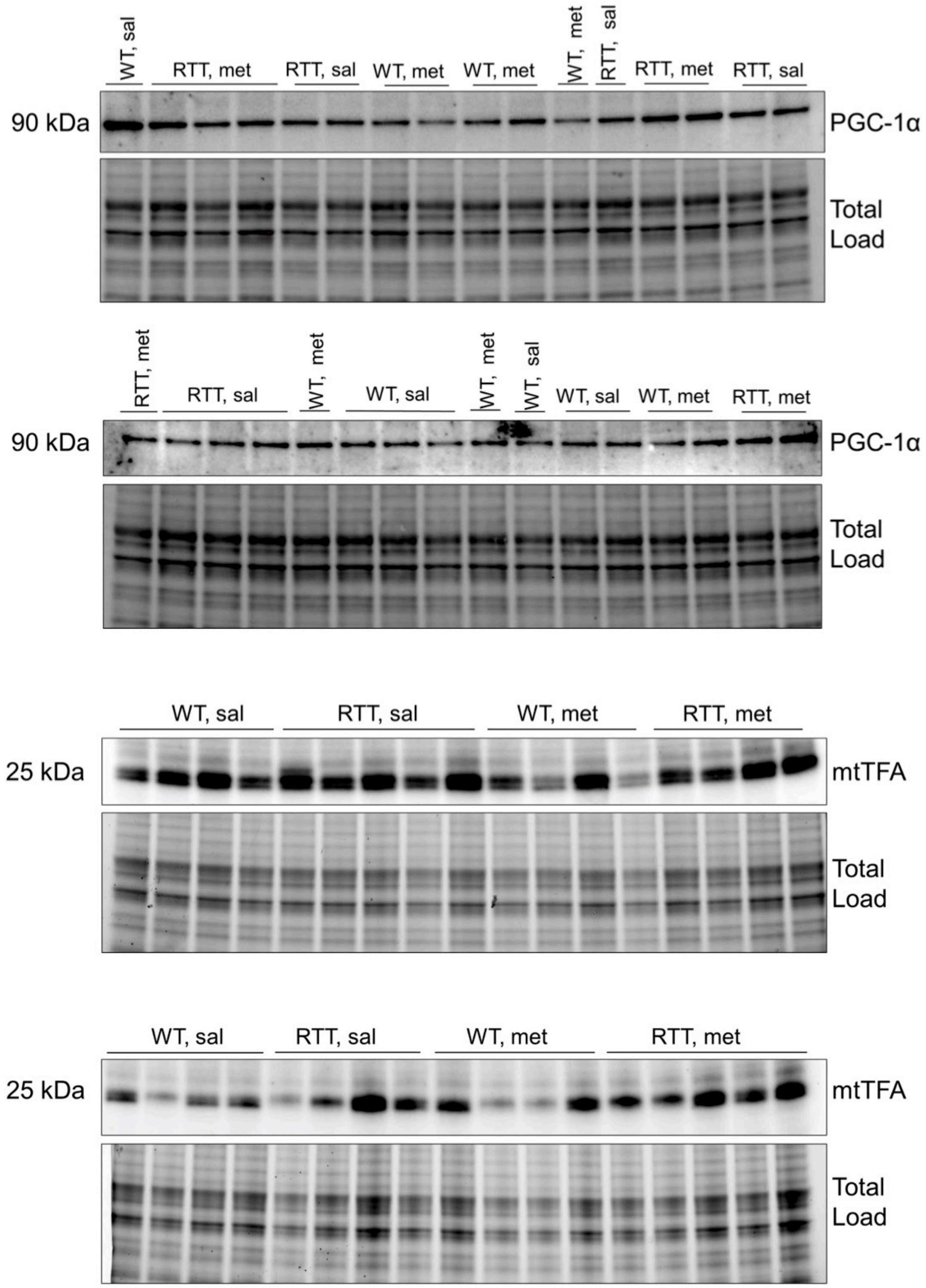

WT, sal
RTT, met
RTT, sal
WT, met
WT, met
WT, met
RTT, sal
RTT, met
RTT, sal
90 kDa
PGC-1α
Total Load
RTT, met
RTT, sal
WT, met
WT, sal
WT, met
WT, sal
WT, sal
WT, met
RTT, met
90 kDa
PGC-1α
Total Load
WT, sal
RTT, sal
WT, met
RTT, met
25 kDa
mtTFA
Total Load
WT, sal
RTT, sal
WT, met
RTT, met
25 kDa
mtTFA
Total Load

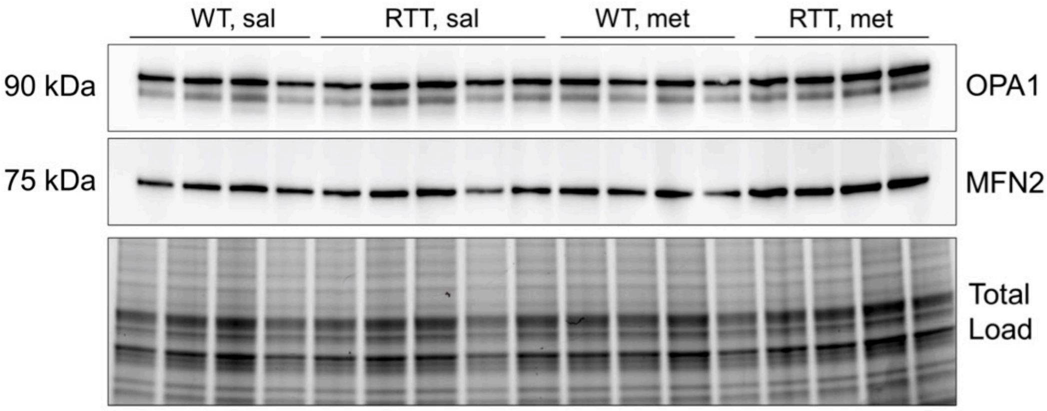

WT, sal
RTT, sal
WT, met
RTT, met
90 kDa
OPA1
75 kDa
MFN2
Total Load

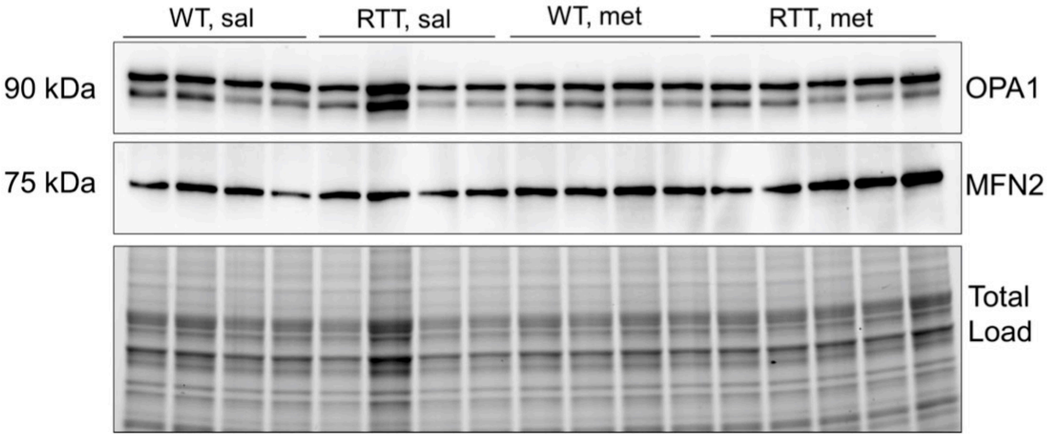

WT, sal
RTT, sal
WT, met
RTT, met
90 kDa
OPA1
75 kDa
MFN2
Total Load

(d)

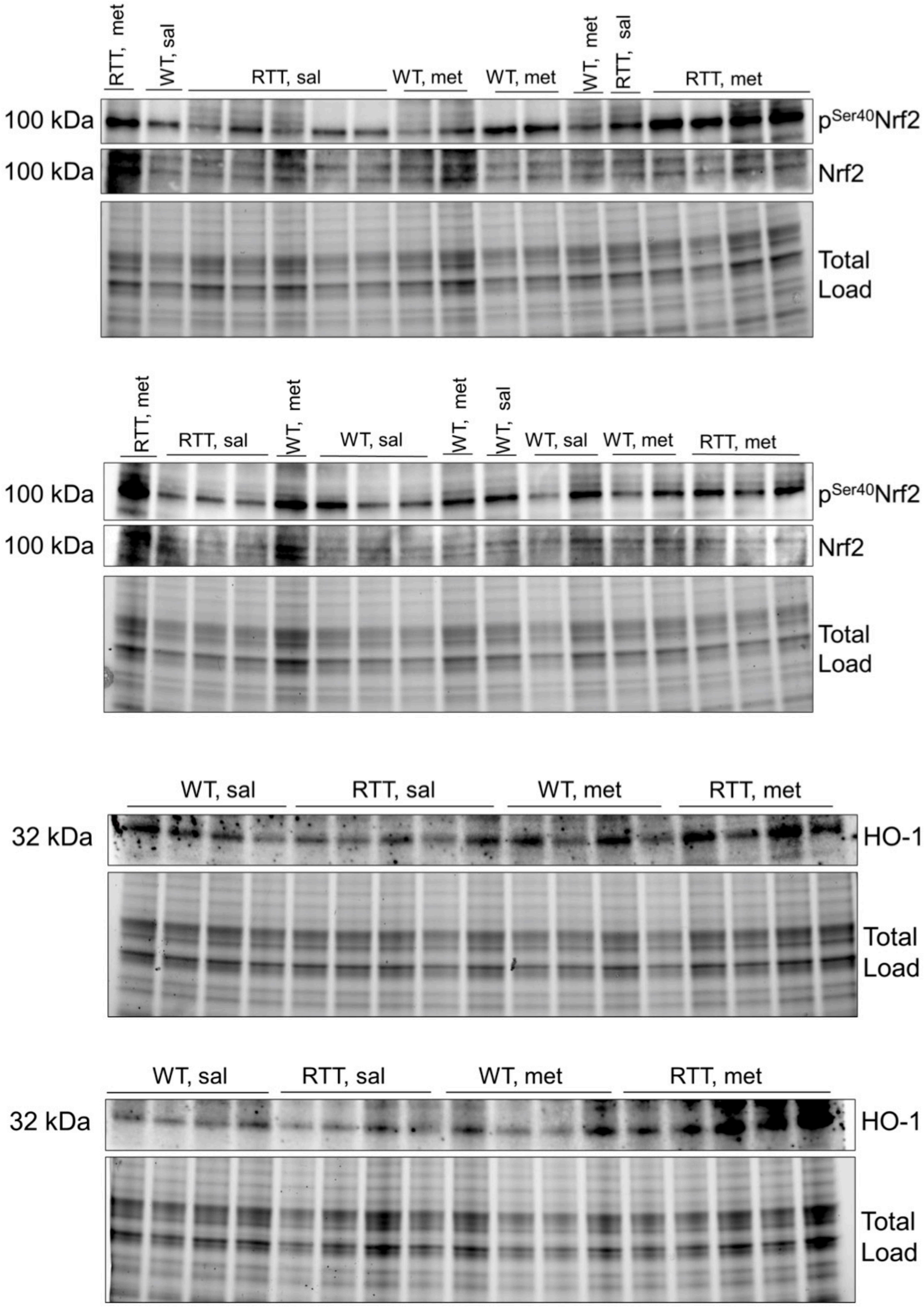

Figure S1 – Full Immunoblot of Western blot results.

The full-lenght blot of (a) Figure 3a, (b) Figure 4b, (c) Figure 5a, (d) Figure 6a. Abbreviations. WT: wild-type mice; RTT: MeCP2-308 heterozygous female mice; sal: saline; met: metformin; NDUFB8: NADH:Ubiquinone Oxidoreductase Subunit B8; SDHB: Succinate Dehydrogenase Complex Iron Sulfur Subunit B; UQCRC2: Ubiquinol-Cytochrome C Reductase Core Protein II; MTCO1: mitochondrially encoded cytochrome c oxidase

I; ATP5A: ATP Synthase, H^+ Transporting, Mitochondrial F1 Complex; HNE-adducts: 4-hydroxy-2-trans-nonenal protein bound; PGC-1α: peroxisome proliferator-activated receptor gamma coactivator 1-alpha; mtTFA: Mitochondrial transcription factor A; OPA1: GTPase Optic Atrophy 1; MFN2: GTPases Mitofusin 2; Nrf2: nuclear respiratory factor 2; HO-1: heme oxygenase-1.
